# Abdominal Epilepsy: A Rare Diagnosis Behind Recurrent Abdominal Pain and Transient Loss of Consciousness

**DOI:** 10.7759/cureus.98364

**Published:** 2025-12-03

**Authors:** Ye Lin Aung, Than T Aye, May Nyein Oo, Htet Yu Ya Oo

**Affiliations:** 1 Haematology, The Clatterbridge Cancer Centre, Liverpool, GBR; 2 Diabetes and Endocrinology, Grand Hantha International Hospital, Yangon, MMR; 3 Geriatrics, Leeds Teaching Hospitals NHS Trust, Leeds, GBR; 4 Neuroscience, University Hospital Southampton NHS Foundation Trust, Southampton, GBR

**Keywords:** abdominal epilepsy, carbamazepine therapy, electroencephalogram, focal seizures, frontal lobe epilepsy, unexplained abdominal pain, visceral pain

## Abstract

Abdominal epilepsy is a rare and under-recognized form of epilepsy that primarily presents with gastrointestinal manifestations such as recurrent abdominal pain, sometimes accompanied by transient loss of consciousness. Because of its atypical presentation, abdominal epilepsy is frequently misdiagnosed, leading to unnecessary investigations and delayed treatment. We report a 47-year-old woman with a 15-year history of recurrent cramping abdominal pain associated with an intense urge to defecate, collapse, and brief loss of consciousness. Multiple gastrointestinal and cardiac evaluations were unremarkable. Neurological assessment and electroencephalography (EEG) demonstrated focal epileptiform discharges in the left frontal region, consistent with focal seizure activity. Treatment with carbamazepine 200 mg twice daily produced marked symptom improvement within one month, and the patient remained asymptomatic at the 12-month follow-up. This case illustrates the diagnostic challenge abdominal epilepsy poses when symptoms mimic gastrointestinal or syncopal disorders. Early EEG evaluation and neurological referral in patients with recurrent, unexplained abdominal pain and episodic altered consciousness can prevent unnecessary procedures and significantly improve outcomes.

## Introduction

Epilepsy is a neurological disorder characterized by recurrent, unprovoked seizures. While most patients present with convulsions or altered awareness, some exhibit atypical manifestations. Abdominal epilepsy is one such uncommon subtype, characterized by paroxysmal abdominal pain that may be accompanied by confusion, dizziness, or transient loss of consciousness [1-4].

Abdominal epilepsy is believed to arise from seizure activity in brain regions that govern visceral sensation, typically the temporal or frontal lobes [2-4]. Because of its rarity and nonspecific presentation, patients often undergo extensive gastrointestinal investigations before neurological causes are considered. The absence of standardized diagnostic criteria further contributes to misdiagnosis as functional abdominal pain [1,4].

Epidemiology and diagnostic delay

Abdominal epilepsy is rare, accounting for <1% of epilepsy cases in published series, and reported diagnostic delays commonly range from two to five years, largely due to atypical presentation and frequent misattribution to gastrointestinal or functional disorders [1-5]. Increasing awareness may reduce unnecessary testing and expedite effective therapy.

We report a case of a middle-aged woman with long-standing unexplained abdominal pain and episodic collapse who was ultimately diagnosed with abdominal epilepsy through electroencephalography (EEG). This case emphasizes the importance of maintaining a broad differential diagnosis and pursuing early neurological assessment when recurrent gastrointestinal symptoms remain unexplained.

## Case presentation

A 47-year-old woman presented with a 15-year history of sudden, stereotyped episodes of cramping abdominal pain lasting several minutes, occasionally accompanied by an intense urge to defecate, collapse with brief loss of consciousness, and rapid spontaneous recovery. Episodes were spontaneous and unrelated to meals or exertion. There were no associated abnormal limb movements, dystonia, automatisms, fever, vomiting, diarrhoea, or persistent bowel habit change. Interictal neurological examination was normal.

Laboratory tests (full blood count, liver and renal profiles, inflammatory markers) were normal. Screening for porphyria/metabolic causes was negative. Abdominal ultrasound showed no abnormality. Computed tomography (CT) of the brain was unremarkable. Cardiac work-up (electrocardiogram (ECG), echocardiography, and 24-hour Holter monitoring) was normal. The patient had previously been labelled as having functional abdominal pain/irritable bowel syndrome (IBS) without benefit.

**Figure 1:**
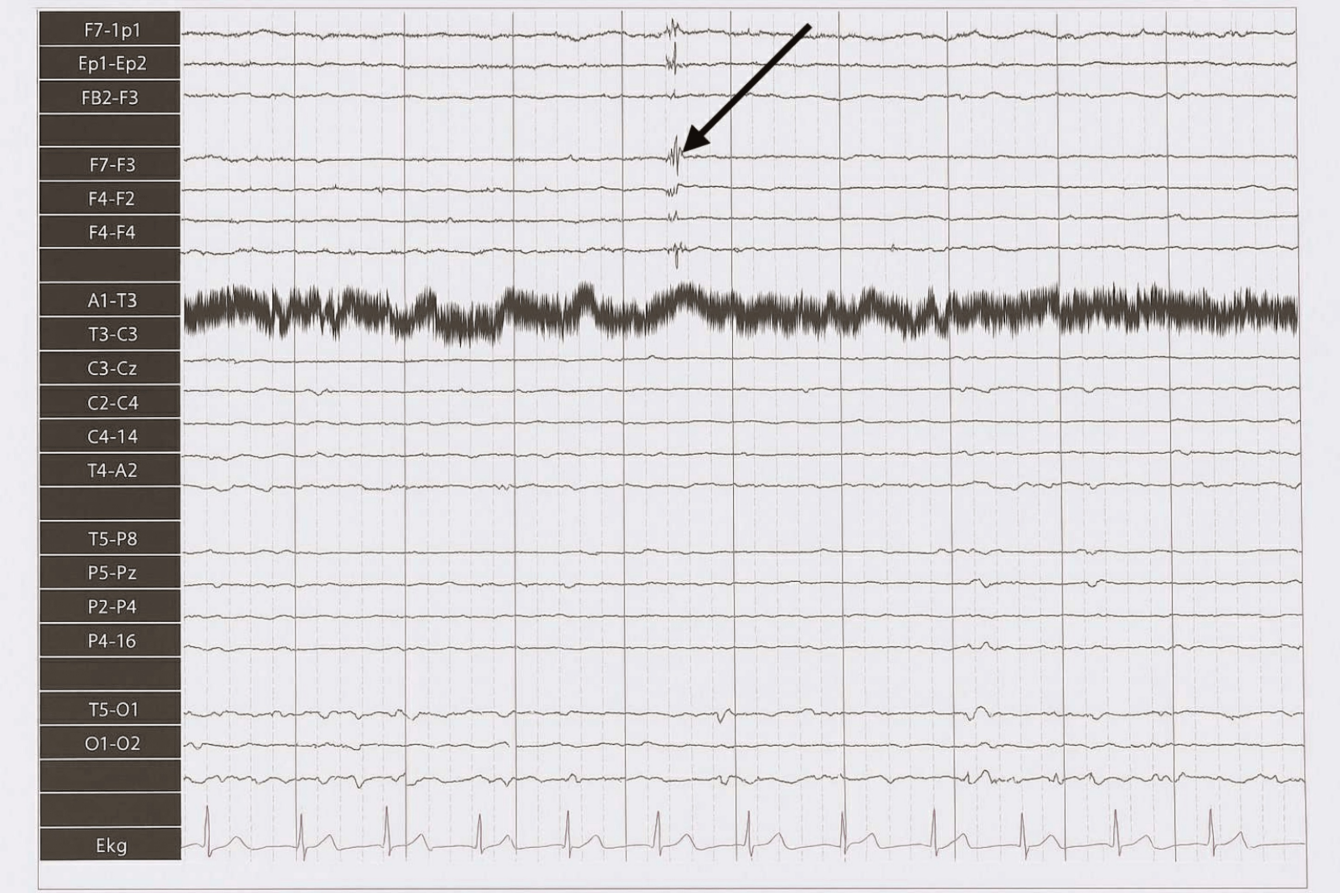
EEG tracing showing focal spike discharges over the left frontal region (Fp1, F7, F4) during drowsiness EEG tracing (10-20 system, average montage) showing focal spike discharges over the left frontal region (Fp1, F7, F4) during drowsiness. This representative segment was obtained from the original printed EEG report (digital file unavailable due to local data access limitations) and was verified by the reporting neurologist. No clinical event was captured during this recording. EEG: electroencephalography

Given the recurrent collapses and negative gastrointestinal/cardiac evaluations, a neurological assessment was pursued. EEG performed using the standard 10-20 international electrode system with an average montage demonstrated focal interictal spike discharges over the left frontal region (Fp1, F7, F4) during drowsiness (Figure 1). No clinical event was captured during the recording.

Carbamazepine 200 mg twice daily, a first-line antiepileptic agent with well-established efficacy in focal-onset seizures [6], was initiated, with a marked reduction in episode frequency within one month and complete symptom resolution sustained at the 12-month follow-up, with no further syncopal events.

A summary of investigations and outcomes is provided in Table 1.

**Table 1:** Timeline of events and investigations GI: gastrointestinal; CT: compute tomography; ECG: electrocardiogram; EEG: electroencephalography

Year	Event/investigation	Result
2007	Onset of episodic abdominal pain	No abnormality found
2009	GI investigations	No pathology identified
2011-2014	Repeated GI + cardiac work-up (ultrasound, CT, ECG, Holter)	Normal
2022	CT of the brain normal; ECG/echo/Holter normal; porphyria/metabolic screening negative: neurology referral + EEG	Focal spikes over the left frontal region
2022	Carbamazepine started	Marked improvement
2023	12-month follow-up	Symptom-free

## Discussion

Abdominal epilepsy is a rare condition in which epileptiform activity manifests predominantly with gastrointestinal symptoms, most commonly paroxysmal abdominal pain. Although more frequently reported in children, adult presentations are well-documented [1-3] but often overlooked due to their nonspecific features. Its rarity and the lack of characteristic gastrointestinal findings frequently lead to misdiagnosis and prolonged diagnostic delay [1,3].

A commonly accepted diagnostic framework includes the following: (1) recurrent abdominal symptoms without an identifiable gastrointestinal cause; (2) evidence of central nervous system disturbance; (3) EEG abnormalities consistent with epilepsy; and (4) clinical improvement with antiepileptic therapy [4].

Our patient satisfied all four elements. Despite years of gastrointestinal and cardiac investigations, no organic cause was identified, prompting neurological referral. EEG demonstrated focal interictal epileptiform discharges over the left frontal region during drowsiness, and antiseizure therapy was followed by sustained symptom resolution at 12 months.

Epileptiform discharges originating in the temporal, insular, or frontal lobes can generate abdominal sensations and autonomic disturbances via limbic and viscerosensory pathways [2-4]. Although temporal lobe epilepsy is more common, frontal lobe involvement, as in this case, can also produce brief paroxysmal abdominal symptoms with transient loss of consciousness. The left frontal spikes observed during drowsiness support this localization.

Alternative causes of episodic abdominal pain were systematically evaluated and considered unlikely: gastrointestinal and cardiac work-ups (including ultrasound, ECG, echocardiography, and Holter monitoring) were normal; CT of the brain showed no structural abnormality; and laboratory testing, including inflammatory markers, metabolic panels, and porphyria screening, was unremarkable. Paroxysmal movement disorders were considered but were not supported by the clinical picture (no dystonia, choreoathetoid movements, or action/startle triggers; normal interictal neurological examination), and unlike epileptic events, these disorders typically occur without loss of consciousness [7].

Two methodological caveats warrant emphasis. First, no electroclinical event was captured during the EEG, and interictal abnormalities can occasionally occur in individuals without clinical epilepsy; therefore, EEG findings should be interpreted in a clinical context. Second, carbamazepine's analgesic and neuropathic pain-modulating properties [8] mean that therapeutic response, while supportive, is not definitive proof of causality. Taken together, the combination of stereotyped paroxysmal episodes, left frontal interictal spikes, comprehensive exclusion of alternative aetiologies, and sustained response to antiseizure therapy makes abdominal epilepsy the most plausible diagnosis in this patient. Where feasible, prolonged video-EEG monitoring (ideally during hospitalization and, if appropriate, with supervised medication withdrawal) could further strengthen diagnostic certainty [9].

This case underscores the importance of considering abdominal epilepsy in patients with recurrent, unexplained abdominal pain, particularly when episodes are stereotyped, associated with collapse or transient loss of consciousness, or resistant to standard gastrointestinal evaluation. Early neurological assessment can reduce unnecessary investigations and improve outcomes [1-5].

## Conclusions

Abdominal epilepsy, although rare, should be considered in patients presenting with recurrent, unexplained abdominal pain and transient neurological symptoms. When standard gastrointestinal and cardiac evaluations fail to identify a cause, early neurological referral and EEG assessment can facilitate timely diagnosis. In this case, abdominal epilepsy represents the most plausible explanation, supported by focal interictal epileptiform discharges and a sustained response to antiseizure therapy, although the absence of electroclinical correlation and the potential analgesic effects of carbamazepine are acknowledged as confounders. Where available, prolonged video-EEG monitoring could further strengthen diagnostic certainty. Prompt recognition and treatment may prevent prolonged morbidity and unnecessary investigations. Greater clinical awareness of this uncommon presentation can enhance recognition and management in both acute and outpatient settings.
